# Effects of foliar spray of agricultural grade mineral oil in springtime, in combination with potassium and calcium sulfates on the phenological and biophysical indices of clusters, and foliar nutritional levels in grapevine (*Vitis vinifera* L.) cv. Sultana (Id. Thompson seedless, Sultanina)

**DOI:** 10.1186/s40659-021-00353-3

**Published:** 2021-09-08

**Authors:** Rouhollah Karimi, Abbas Saberi, Ali Khadivi

**Affiliations:** 1 Department of Horticulture and Landscape Engineering, Faculty of Agriculture, Malayer University, Malayer, Iran; 2 Department of Horticultural Sciences, Faculty of Agriculture and Natural Resources, Arak University, 38156-8-8349 Arak, Iran

**Keywords:** Grape, Fruit quality, Mineral oil, Potassium, Calcium

## Abstract

**Background:**

Improving the nutritional condition of grapevine in spring to regulate bloom, fruit set, and yield is among the management goals of vineyards.

**Methods:**

In the present study, the early season spray of calcium sulfate (C; 0.00 and 2.00%), potassium sulfate (K; 0.00 and 3.00%), and agricultural grade mineral oil (V; 0.00 and 1.00%) on flower and fruit phenology, nutrient concentration, and cluster biophysical indices and yield of Sultana grapevine (*Vitis vinifera* L.) were investigated for two consecutive years.

**Results:**

Based on the results, the spray of this nutrient combined with mineral oil significantly affected all the treatments except cluster length, berry length, and phosphorus concentration. The highest concentrations of potassium, calcium, and magnesium were obtained in the vines treated with V_0_K_1_C_1_, and the highest concentrations of zinc and iron were obtained only in the vines treated with mineral oil. In treatments containing mineral oil, especially in combination with the second level of calcium and potassium (V_1_K_1_C_1_), bloom time, berries pea-sized time, and harvest time were delayed by 3, 3, and 6 days compared with control vines. While in vines treated with a combination of the second level of potassium and calcium (V_0_K_1_C_1_), bloom time, berries pea-sized time, and harvest time were advanced by 5, 4, and 1.50 days, respectively, compared with control vines. Regarding the biophysical indices of the cluster, it was found that the vines treated with V_1_K_1_C_1_ had higher cluster weight, berry weight, fruit, and raisins yield than other treatments. Also, the highest berry quality, including total soluble solids, titratable acidity, and total phenol content, were obtained in the vines treated with V_0_K_1_C_1_. However, the lowest berry quality was observed in the vines treated with mineral oil.

**Conclusions:**

Therefore, the combination of nutrients with mineral oil can alleviate the adverse effect of mineral oil solely on some phenological indices and berry quality-related traits in vineyards.

## Background

Achieving the right fertilizer composition to produce a sustainable and high-quality product in vineyards is one of the main viticultural management programs. In this regard, every year, a significant part of chemical fertilizers is given to the vineyards at the non-appropriate time or amounts which not only do not increase yield and quality of vines but also imposes high costs on the vine-growers and leads to soil pollution and salinity. Most of the nutritional needs of trees are added to the soil in the form of organic and chemical fertilizers, but due to barriers to solubility and ion uptake, only a small part of these elements is transferred to the aerial parts of the tree [32]. These limitations can affect negatively both growth and development of fruits especially when there is a greater need for nutrients. At the beginning of growing season, due to increased growth in buds and the predominance of vegetative growth, the concentration of nutrients decreases dramatically in vines tissues [26, 30]. Therefore, supplemental application of these elements at the beginning of growing season along with mineral oils may improve the concentration of these elements in tissues and trigger metabolic activities related to these elements, which ultimately leads to increased yield and fresh fruit quality.

Various horticultural mineral oils have been used in recent years to delay the budbreak or bloom time in fruit trees [9]. In this regard, soybean oil in almonds [7], pistachios [23], and grapes [11, 46], by preventing CO_2_ emissions and reducing respiration in buds, successfully delayed budburst in treated plants. Horticultural mineral oil has always been considered to be a dormant oil to eliminate chilling requirement of trees and to control insects and mites [7, 19]. It could be also mixed with plant nutrients to increase their absorption and affect leaf nutritional status [1, 9]. The use of mineral oil in winter simultaneously with full dormancy stage of buds has led to accelerated blooming, increased flowering uniformity, and increased quantity and quality of pistachio nuts [9, 23]. However, its use after the ecodormancy stage has delayed budbreak [7, 11, 29]. However, untimely or high concentrations of mineral oil may cause bud necrosis rate, delayed fruit ripening, and reduced fruit quality and production of raisins, number of clusters, and yield in grapes [12, 46].

The use of nutrients in combination with mineral oils with the aim of osmotic regulation in the bud and with the aim of repairing possible damage caused by mineral oils is one of the management methods which may affect the ripening time and quality of the fruit by accelerating the phenological stages of the fruit and completing the optimal growth. Calcium and potassium are among the essential elements that due to their structural and enzymatic roles can affect endogenous hormones, soluble sugar content, budburst time, and final yield of fruit trees [24, 26]. Calcium is involved in the binding of polysaccharides and proteins that make up the cell wall [32]. It also responds to environmental signals and hormones as a secondary messenger in plants [5]. This element contributes to auxin activity and is involved in cell division and cell elongation, germination, and pollen tube growth [21]. Calcium is also effective in improving flowering, maturation, and transfer of carbohydrates from leaves to fruits [32]. Potassium is involved in many aspects of metabolism such as photosynthesis, protein, and sugar transport, activation of more than 60 enzymes, regulation of osmotic potential, regulation of stomata, and phloem formation [24, 32]. Potassium deficiency stops the transfer of sugar in the phloem vessels and can cause sucrose accumulation in the leaves to compensate reduced osmotic potential caused by its deficiency [34]. It has been shown that the use of potassium improved cluster weight, berry color, yield, and fruit internal quality characteristics [20, 24, 48]. Most of the qualitative characters of berry and seed of grape are affected by nutrients [2, 33, 37–39].

Despite the separate use of potassium, calcium, and mineral oil in the previous studies, the combined use of these substances on bloom time, fruit set, fruit quantitative and qualitative indices, and also leaf mineral nutrients content has not been reported in fruit trees. On the other hand, the previous studies have shown that the use of calcium sources alone can reduce the amount of potassium in leaves and fruits in different trees, which highlights the need for combined use of calcium and potassium [25, 44]. Therefore, in the present study, the combined and separate effects of these compounds on phenological changes in Sultana grape cultivar in vineyard conditions were evaluated. The ultimate goal of this study was to achieve the appropriate combination of fertilizers with mineral oil and to investigate its effects on phenological characters (the times of budburst, bloom, and ripening), fruit set percentage, yield components (number of clusters in vine, berry weight, cluster weight, fruit yield, and raisins) and some fruit quality indices (soluble solids, titratable acidity, pH, ascorbic acid, and total phenol content) as well as the concentration of elements in the leaves. The special goal of this study was to investigate the efficiency of potassium and calcium application in reducing the negative effects of mineral oil, especially on the sugar content and fruit ripening time in Sultana grapevine cultivar.

## Materials and methods

### Plant material and treatments

The present experiment was accompanied in 2017 and 2018, on the own root of 20-year-old grapevines (*V. vinifera* cv. ‘Sultana’) from a commercial vineyard at Abbas-Abad village of Khondab (Lat. 34˚38′N, Long. 49˚10′E, and Alt. 1707 m) in Markazi province/Iran. Minimum, maximum, and mean annual temperatures of Khondab area were − 16.50 °C, 38.40 °C, and 13.20 °C, respectively, with mean rainfall per annum of 318 mm. Although this site has relatively long and hot summers, the winter temperatures may plunge down to − 17 °C or even lower. The vineyard site has loamy soil; some physicochemical parameters of soil at the commercial vineyard are shown in Table 1.

**Table 1:** Analysis of soil samples at experimental vineyard

Soil depth (cm)	Clay (%)	Sand (%)	Silt (%)	Soil texture	pH	EC (ds/m)	T.N.V **(%)**	Absorbable P (ppm)	Absorbable K (ppm)	Total N **(%)**	Total C **(%)**
0–30	36.20	26.40	37.40	CL	7.70	0.77	19	19	200	0.05	0.52
30–60	35.70	27.80	36.50	CL	7.80	0.76	28	14	167	0.03	0.27

Vines with non-trellised and density of 2 × 4 m were watered under furrow irrigation system and were annually pruned in early March so that every vine had 15 fruit spurs of 6-buds each. The experimental layout was factorial, based on a randomized complete block design with three replications per treatment and two vines per replication. Base mineral fertilizers were applied per hectare at the rates of 130 kg ammonium sulfate, 120 kg triple superphosphate, 150 kg potassium sulfate, and 45 kg magnesium sulfate, and 5 kg zinc sulfate monohydrate, based on soil test interpretations [43].

The vines were sprayed with calcium sulfate (CaSO_4_; 0.00 and 2.00%), potassium sulfate (K_2_SO_4_; 0.00 and 3.00%) and mineral oil (Volck^®^; 0.00 and 1.00%) each at two levels to run-off in 2017 (25 March and 01 April) and 2018 (26 March and 02 April; [13]. Carboxymethylcellulose sodium (CMC; 1.00% W/V) was used as emulsifier to mix water and oil. The different foliar treatments were named as V_0_K_0_C_0_ (control, mineral oil, K_2_SO_4_, and CaSO_4_ at 0.00%), V_0_K_1_C_0_ (0.00% mineral oil, 3.00% K_2_SO_4_, and 0.00% CaSO_4_), V_0_K_0_C_1_ (0.00% mineral oil, 0.00% K_2_SO_4_, and 2.00% CaSO_4_), V_0_K_1_C_1_(0.00% mineral oil, 3.00% K_2_SO_4_, and 2.00% CaSO_4_), V_1_K_0_C_0_ (1.00% mineral oil, 0.00% K_2_SO_4_, and 0.00% CaSO_4_), V_1_K_1_C_0_ (1.00% mineral oil, 3% K_2_SO_4_, and 0% CaSO_4_), V_1_K_0_C_1_ (1.00% mineral oil, 0.00% K_2_SO_4_, and 2.00% CaSO_4_), and V_1_K_1_C_1_ (1.00% mineral oil, 3.00% K_2_SO_4_, and 2.00% CaSO_4_). The vines treated in 2017 were the same treated also in 2018.

### Phenological measurements

In both years, to record phenological indices during the growing season, vines were monitored every day after budbreak and the time of occurrence of different phenological stages was recorded using the Eichhorn-Lorenz (EL) system [18], where higher EL values indicate more advanced phenology [46]. The phenological stages that were recorded in the present study included (1) days to full bloom stage (DFB; EL stage 23), (2) days to berries pea-sized stage (DBP; EL stage 31) and (3) days to commercial ripening stage (DTR; EL stage 38). Likewise, fruit set (EL stage 27) percentage was determined by the ratio of swelled berries to counted florets.

The DFB was recorded as the number of days from the date of second spraying and to the date when the clusters of vines had shown 50% of flower-hoods fallen, while the DBP was counted from the date of second spraying to the date when berry had reached the pea-size stage (unripe berries with pH of 2.50 and TSS of 9). The DTR was counted from the date of second spraying to the date when berries had reached to sugar level with 18 °Brix.

Fruits were harvested in the fourth week of September according to the commercial maturity index of 18 °Brix in control vines and transferred to the Horticultural Research Laboratory of Malayer University to determine the fruit and raisin yield and fruit yield components and to measure the quantitative and qualitative characteristics.

### Fruit biophysical indices and yield

The total number of clusters per vine was recorded as the average number of clusters counted on three selected vines in each treatment at harvest time. The berry length and berry diameter were recorded with a digital caliper as the average length and diameter of 20 randomly selected berries in each treatment at harvest. Moreover, cluster length was recorded with a digital caliper as the average length of five randomly selected clusters in each treatment at harvest. Other biophysical characters such as berry weight and cluster weight were measured with a digital scale. Vine yield was the average weight of harvested clusters on the three selected vines in each treatment.

### Fruit quality indices

In each treatment, berry quality-related traits, including total soluble solids (TSS), titratable acidity (TA), pH, ascorbic acid, and total phenol content were measured in the harvested clusters. The TSS of berry juice was measured at 25 °C using a refractometer (Atago, Japan) and sugar level was expressed as °Brix. Total acidity was determined in the same juice by titration with NaOH (0.10 N) up to pH 8.10, using 1.00 mL of diluted juice in 25 mL distilled H_2_O, and results were expressed as g tartaric acid equivalent per liter.

To measure the ascorbic acid (vitamin C) of fruits, the amount of 1.27 g of iodine was mixed with 16.60 g of potassium iodide in distilled water and its volume was brought to one liter. Then 5.00 ml of the filtered fruit extract was mixed with 20 ml of distilled water and 1.00 ml of 1.00% starch solution was added to it. It was prepared with potassium iodide solution and titrated until dark water appeared and its ascorbic acid content was calculated [6].

The Folin-Ciocalteau method was used to measure total phenol according to Velioglu et al. [45] with some modifications. In total, 500 mg of fresh leaves tissue was mixed with methanol (85%) and after centrifugation at 6000 × g for 10 min, a mixture of 0.30 mL of methanolic extract and one mL Folin-Ciocalteau reagent (10%) was prepared. Five minutes after fast vortexing the tubes, 1.00 mL of sodium carbonate (7%) was mixed with the resulting solution and was placed in a shaker for 20 min at RT in darkness. The total phenolic content of all samples was measured at 765 nm. Finally, by means a standard curve preparing by gallic acid, total phenolic content was determined and the results were represented as mg per gram of fresh weight (FW).

### Raisin preparation

Chemical pre-drying treatments that hasten drying were applied for raisin processing. Briefly, 5.00 kg of freshly grape clusters were washed carefully with tap water and dipped into an alkaline oil-in-water emulsion known to grapevine growers as ‘cold dip’ (50 g potassium carbonate + 2 mL ethyl oleate + 1L distilled water) for 5 min [28, 40]. After this treatment, grape clusters were spread on a drying rack with wire mesh sheets under direct sunlight and left on the rack until they reach 15% moisture for 5 days [28]. After drying, the resulting raisins were boxed immediately after shaking and kept in a cool place. Yield of raisins was calculated by weighing raisins obtained from each kg of grapes in each treatment [3].

### Leaf mineral content

Samples of leaves were collected on 25 July 2017–2018 and their N, P, K, Mg, Ca, Zn, Mn, and Fe concentrations were measured. Three leaves were taken from the middle of growing shoots. Petioles were oven-dried at 70 °C until constant weight, then ground to a powdery texture and 0.20 g was taken to determine the aforementioned elements. Total N was determined by the Kjeldahl. The P was determined using a spectrophotometer. The K was flame photometrically determined. The sample extracts were analyzed for Mg, Ca, Fe, Zn, and Mn using an atomic absorption spectrophotometer (Varian, 220) [20].

### Statistical analyses

Results were subjected to a two-way analysis of variance (ANOVA) using GLM procedures of the SAS software package (SAS Institute Inc., Cary, NC, USA), and mean separation was done using Duncan’s multiple range test at *P* ≤ 0.05.

## Results

### Leaf macro-elements

The effect of calcium, potassium, and mineral oil and their interaction effects on the content of leaf macro-elements were significant at 0.01 probability level. The nitrogen and calcium content of the leaves in the vines treated with the combination of the second level of potassium sulfate and calcium sulfate without mineral oil (V_0_K_1_C_1_) was higher compared with other treatments (Table 2). Also, the potassium and magnesium content of leaves in the vines treated with the second level of potassium sulfate alone (V_0_K_1_C_0_) was higher than in other treatments (Table 2).

**Table 2:** The combination effect of mineral oil, potassium sulfate, and calcium sulfate on leaf macro-elements of Sultana grapevine

Treatment	N (%)		K (%)		P (%)		Ca (%)		Mg (%)	
	2017	2018	2017	2018	2017	2018	2017	2018	2017	2018
V_0_K_0_C_0_	2.26 b	2.07 c	1.35 e	1.31 e	0.38 b	0.35 b	2.07 cd	2.01 d	1.13 c	1.20b c
V_0_K_1_C_0_	2.41 a	2.33 ab	2.04 a	2.16 a	0.28 c	0.35 bc	2.58 b	2.67 b	1.50 a	1.35 a
V_0_K_0_C_1_	2.38 a	2.37 a	1.74 bc	1.72 c	0.40 a	0.39 ab	2.63 b	2.70 b	1.30 b	1.25 b
V_0_K_1_C_1_	2.45 a	2.43 a	1.84 b	1.97 bc	0.42 a	0.30 c	2.84 a	2.87 a	1.45 ab	1.17 ab
V_1_K_0_C_0_	2.03 c	1.72 d	1.48 de	1.56 d	0.43 a	0.45 a	2.00 d	1.92 d	1.13 d	1.16 cd
V_1_K_1_C_0_	2.07 c	2.03 c	1.90 ab	2.03 a	0.35 b	0.30 c	2.36 cd	2.40 c	1.22b c	1.17 cd
V_1_K_0_C_1_	2.25 b	2.22 b	1.61 cd	1.79 c	0.35 b	0.35 b	2.45 bc	2.70 b	1.13 c	1.06 d
V_1_K_1_C_1_	2.35 ab	2.22 b	1.99 a	2.10 ab	0.38 ab	0.33 bc	2.47 b	2.69 b	1.43 ab	1.28 ab

The means with common letters in each column according to Duncan's test are not significantly different (*P* < 0.05). V_0_ = 0.00% mineral oil, V_1_ = 1.00% mineral oil; K_0_ = 0.00% K_2_SO_4_, K_1_ = 3.00% K_2_SO_4_; C_0_ = 0.00% CaSO_4_, C_1_ = 2.00% CaSO_4_

Leaf phosphorus content in the vines treated with mineral oil alone (V_1_K_0_C_0_) was higher than in other treatments (Table 2). The lowest content of nitrogen, calcium, and magnesium of the leaves was related to the vines treated with mineral oil alone (Table 2). Control vines (V_0_K_0_C_0_) showed less potassium compared with other treatments (Table 2). The vines treated with potassium (V_0_K_1_C_0_) in 2017 and also the vines treated with potassium sulfate and calcium sulfate (V_0_K_1_C_1_) in 2018 showed lower phosphorus content (Table 2).

### Leaf micro-elements

The effect of calcium, potassium, and mineral oil and interaction effects on the content of leaf micro-elements were significant at 0.01 probability level. Leaf iron and zinc content in the vines treated with mineral oil alone (V_1_K_0_C_0_) was higher than other treatments, although it did not show a statistically significant difference with some treatments (Table 3).

**Table 3:** The combination effect of mineral oil, potassium sulfate, and calcium sulfate on leaf micro-elements of Sultana grapevine

Treatment	Fe (ppm)		Zn (ppm)		Mn (ppm)	
	2017	2018	2017	2018	2017	2018
V_0_K_0_C_0_	116.3 d	118.5 d	54.52 c	55.46 cd	111.5 c	112.6 c
V_0_K_1_C_0_	125.1 cd	120.6 c	59.53 b	58.40 c	120.2 b	123.1 b
V_0_K_0_C_1_	112.3 d	115.5 d	53.50 c	55.05 cd	112.7 c	117.5 bc
V_0_K_1_C_1_	121.5 d	130.1 b	52.30 c	51.10 d	133.3 a	136.5 a
V_1_K_0_C_0_	148.0 a	146.5 a	69.87 a	68.62 a	112.2 c	116.3 bc
V_1_K_1_C_0_	144.7 ab	143.6 a	68.15 a	67.21 a	117.2 bc	120.7 b
V_1_K_0_C_1_	132.2 abc	135.70 b	61.88 b	63.45 b	120.5 b	120.2 b
V_1_K_1_C_1_	140.2 abc	145.9 a	58.22 b	67.55 ab	124.3 b	125.3 b

The means with common letters in each column according to Duncan's test are not significantly different (*P* < 0.05). V_0_ = 0.00% mineral oil, V_1_ = 1.00% mineral oil; K_0_ = 0.00% K_2_SO_4_, K_1_ = 3.00% K_2_SO_4_; C_0_ = 0.00% CaSO_4_, C_1_ = 2.00% CaSO_4_

Vines treated with mineral-free calcium sulfate and potassium sulfate (V_0_K_1_C_1_) showed higher leaf manganese content than other treated compounds (Table 3). The lowest leaf iron content was observed in the vines treated with calcium sulfate treatment alone (V_0_K_0_C_1_), while the lowest leaf zinc was in the vines treated with calcium sulfate and potassium sulfate (V_0_K_1_C_1_) and the lowest manganese was in control vine leaves (V_0_K_0_C_0_) (Table 3).

### Phenological indices

Flowering time (EL stage 23) in the vines treated with the combination of the second level of calcium, potassium, and mineral oil (V_1_K_1_C_1_) (without significant difference with mineral treatment alone) occurred with a longer delay compared with other treatments (Table 4). In contrast to the second level treatment of calcium sulfate and potassium sulfate (V_0_K_1_C_1_), the flowers bloomed faster than other treatments, so it did not show statistically significant differences with the flowering time in the vines treated with the second level of potassium sulfate alone (Table 4).

**Table 4:** The combination effect of mineral oil, potassium sulfate, and calcium sulfate on phenological indices of Sultana grapevine

Treatment	Days to full bloom stage		Days to berries pea-sized stage		Days to ripening stage		Fruit set (%)	
	2017	2018	2017	2018	2017	2018	2017	2018
V_0_K_0_C_0_	54.2 b	53.4 c	69.0 b	68.50 b	138.5 b	141.50 ab	24.2 cd	24.4 c
V_0_K_1_C_0_	50.1 cd	49.4 d	65.5 c	64.50 c	137.0 b	137.50 c	31. 5 a	35.5 a
V_0_K_0_C_1_	51.0 c	51.1 d	66.5 c	66.50 bc	137.5 b	139.50 bc	27.4 bcd	26.4 bc
V_0_K_1_C_1_	49.5 d	50.3 d	65.0 c	65.50 c	137.0 b	138.50 bc	28.8 ab	30.8 ab
V_1_K_0_C_0_	57.0 a	56.9 a	72.5 a	72.50 a	144.5 a	145.50 a	25.5 cd	24.50 c
V_1_K_1_C_0_	55.5 ab	54.8 b	71.5 a	72.50 a	143.0 a	145.50 a	28.3 abc	28.0 b
V_1_K_0_C_1_	55.0 ab	56.2 a	71.5 a	72.50 a	143.0 a	145.50 a	27.7 bc	28.5 b
V_1_K_1_C_1_	56.5 a	56.7 a	71.5 a	72.50 a	143.5 a	145.50 a	28.7 abc	31.2 ab

The means with common letters in each column according to Duncan's test are not significantly different (*P* < 0.05). V_0_ = 0.00% mineral oil, V_1_ = 1.00% mineral oil; K_0_ = 0.00% K_2_SO_4_, K_1_ = 3.00% K_2_SO_4_; C_0_ = 0.00% CaSO_4_, C_1_ = 2.00% CaSO_4_

Berries pea-sized phase (EL stage 31) as an important stage grape development was affected significantly by the use of potassium sulfate, calcium sulfate, and mineral oil. Interestingly, the berries pea-sized stage was reached 6 days later in the vines treated with mineral oil at 1.00% with or without calcium sulfate and potassium sulfate compared with vines sprayed with the second level of calcium sulfate and potassium sulfate without mineral oil (Table 4). However, the vines treated with mineral oil with and without calcium sulfate and potassium sulfate were different from control vines (V_0_K_0_C_0_) in both years for 2 to 3 days until reaching the berries pea-sized stage (Table 4).

Harvest time (EL stage 38) based on commercial ripening stage index (˚Brix) was affected by the use of mineral oil in combination with calcium sulfate and potassium sulfate. Application of mineral oil with or without calcium sulfate and potassium sulfate delayed fruit ripening stage compared with control vines. However, the combination of potassium sulfate and calcium sulfate without mineral oil (V_0_K_1_C_1_) accelerated fruit ripening stage compared with control vines (Table 4).

Mineral oil spray in combination with calcium sulfate and potassium sulfate significantly affected fruit set (EL stage 27) percentage. Fruit formation in the vines treated with the second level of potassium sulfate (V_0_K_1_C_0_) increased by 23.20% compared with control vines. There was no significant difference between the other treatments in terms of fruit formation (Table 4).

### Yield components

Cluster number, cluster weight, cluster length, berry weight, and berry length were significantly affected by mineral oil spray in combination with calcium sulfate and potassium sulfate. The number of clusters in the vines treated with the second level of mineral oil alone was the highest in 2017 and in the vines treated with the second level of calcium alone in 2018, however, it did not show a significant difference with many treatments in this regard (Table 5).

**Table 5:** The combination effect of mineral oil, potassium sulfate, and calcium sulfate on cluster characteristic and yield components of Sultana grapevine

Treatments	Cluster number (no.)		Cluster weight (g)		Cluster length (cm)		Berry weight (g)		Berry length (cm)	
	2017	2018	2017	2018	2017	2018	2017	2018	2017	2018
V_0_K_0_C_0_	73.3 ab	72.4 b	163.5 cd	164.9 d	18.9 b	18.4 b	0.84 d	0.76 d	1.46 c	1.48 b
V_0_K_1_C_0_	67.8 c	69.4 bc	196.2 a	195.3 ab	21.6 a	20.3 ab	1.25 a	1.28 a	1.71 a	1.65 a
V_0_K_0_C_1_	71.8 abc	75.1 a	171.2	175.6 cd	20.2 ab	21.5 a	0.99 bc	0.87 cd	1.58 bc	1.54 ab
V_0_K_1_C_1_	67.6 c	67.7 c	191.6 a	200. 6 a	19.4 b	21.5 a	1.16 ab	0.95 c	1.47 bc	1.44 b
V_1_K_0_C_0_	75.9 a	76.3 a	153.5e	152.9e	18.2 b	19.0 b	0.84 d	0.92 c	1.44 c	1.41 bc
V_1_K_1_C_0_	70.3 abc	71.4 b	180.9 b	182.3 c	21.2 a	22.2 a	1.05 b	1.15 ab	1.54 bc	1.35 c
V_1_K_0_C_1_	74.7 ab	76.5 a	158.5ed	157.9 de	21.8 a	21.9 a	0.89 cd	0.92 c	1.55 bc	1.32 c
V_1_K_1_C_1_	69.5 bc	72.1 b	187.8 ab	192.6 b	21.9 a	19.0 b	1.11 b	1.10 b	1.57 bc	1.48 b

The means with common letters in each column according to Duncan's test are not significantly different (*P* < 0.05). V_0_ = 0.00% mineral oil, V_1_ = 1.00% mineral oil; K_0_ = 0.00% K_2_SO_4_, K_1_ = 3.00% K_2_SO_4_; C_0_ = 0.00% CaSO_4_, C_1_ = 2.00% CaSO_4_

In both years, the weight of the clusters of the vines treated with the second level of potassium alone (V_0_K_1_C_0_; in 2017) or the combined treatment of the second level of potassium and calcium (V_0_K_1_C_1_; in 2018) was more than the other treatments (Table 5). Also, in the study of treatments, it was found that the application of the second level of mineral oil reduced the weight of the clusters in comparison with the first level treatments of mineral oil (treatments without the use of mineral oil). For instance, the weight of the panicle in the potassium sulfate treatment alone was 196.20 g in 2017, but the weight of the cluster in the vines treated with this treatment and mineral oil was 180.90 g (Table 5).

Application of the second level of potassium sulfate and calcium sulfate with and without combination with mineral oil had better effects on the length of the clusters compared with the control and vines treated with the second level of mineral oil alone (Table 5). The weight and length of berries in the vines treated with the second level of potassium sulfate alone (V_0_K_1_C_0_) was higher than in other treatments. The lowest weight and length of berries without significant differences were observed in the control vines and the vines treated with the second level of mineral oil alone (Table 5).

**Fig. 1:**
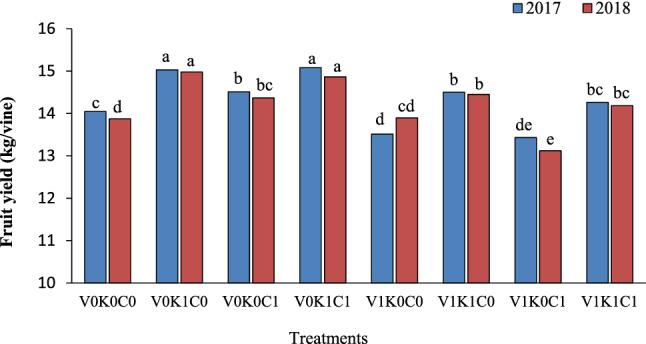
The combination effect of mineral oil, potassium sulfate, and calcium sulfate on yield of Sultana grapevine. The means with common letters in each column according to Duncan's test are not significantly different (*P* < 0.05). V_0_ = 0.00% mineral oil, V_1_ = 1.00% mineral oil; K_0_ = 0.00% K_2_SO_4_, K_1_ = 3.00% K_2_SO_4_; C_0_ = 0.00% CaSO_4_, C_1_ = 2.00% CaSO_4_

The yield of the vines treated with the combination of the second level of potassium sulfate and calcium sulfate without mineral oil (V_0_K_1_C_1_) was higher than in other treatments. However, there was no significant difference with the yield of the vines treated with potassium sulfate level alone in this regard (Fig. 1). The lowest yield was in the vines treated with the second level of mineral oil in combination with calcium sulfate.

### Qualitative indices

Berries pH, TA, and TSS were affected by the treatment of mineral oil in combination with calcium sulfate and zinc sulfate. The pH and TSS of berries in the vines treated with the combined treatment of the second level of calcium sulfate and potassium sulfate without mineral oil (V_0_K_1_C_1_) were higher compared with other treatments. The lowest berries pH and TSS were in the control vines and the vines treated with the second level of mineral oil alone (Table 6). On the other hand, in the vines treated with mineral alone (V_1_K_0_C_0_), the amount of berries TA was higher compared with other treatments, although there was no statistically significant difference with some treatments (Table 6).

**Table 6:** The combination effect of mineral oil, potassium sulfate, and calcium sulfate on berry quality characteristic and yield components of Sultana grapevine

Treatments	pH		TA		TSS (˚Brix)	
	2017	2018	2017	2018	2017	2018
V_0_K_0_C_0_	3.38 c	3.38 c	2.54 cd	2.54 bc	18.2 d	17.9 de
V_0_K_1_C_0_	3.49 ab	3.62 a	2.55 ab	2.58 a	22.1 ab	22.4 bcd
V_0_K_0_C_1_	3.40 c	3.40 bc	2.51e	2.54 abc	19.4 cd	19.7 d
V_0_K_1_C_1_	3.55 a	3.65 a	2.53 de	2.53 bcd	24.3 a	25.4 a
V_1_K_0_C_0_	3.35 c	3.37 c	2.57 a	2.58 a	18.9 d	18.8 de
V_1_K_1_C_0_	3.42 bc	3.47 b	2.56 ab	2.56 ab	21.4 bc	23.3 bc
V_1_K_0_C_1_	3.42 bc	3.39 bc	2.56 ab	2.57 a	19.6 bcd	21.5 cd
V_1_K_1_C_1_	3.52 a	3.61 a	2.53 de	2.52 cd	23.9 b	23.8 b

The means with common letters in each column according to Duncan's test are not significantly different (*P* < 0.05). V_0_ = 0.00% mineral oil, V_1_ = 1.00% mineral oil; K_0_ = 0.00% K_2_SO_4_, K_1_ = 3.00% K_2_SO_4_; C_0_ = 0.00% CaSO_4_, C_1_ = 2.00% CaSO_4_

**Fig. 2:**
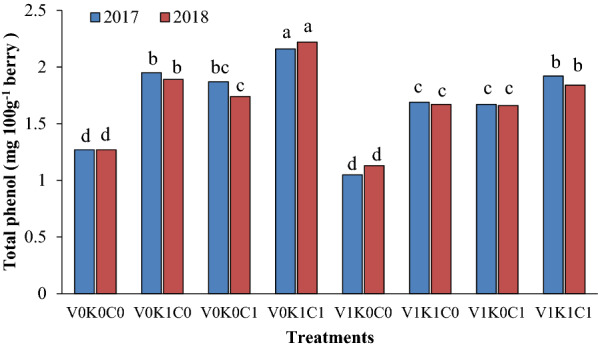
The combination effect of mineral oil, potassium sulfate, and calcium sulfate on berry total phenolcontent of Sultana grapevine. The means with common letters in each column according to Duncan's test are not significantly different (*P* < 0.05). V_0_ = 0.00% mineral oil, V_1_ = 1.00% mineral oil; K_0_ = 0.00% K_2_SO_4_, K_1_ = 3.00% K_2_SO_4_; C_0_ = 0.00% CaSO_4_, C_1_ = 2.00% CaSO_4_

The berries phenol and ascorbic acid content in vines was affected by spray of mineral oil in combination with potassium sulfate and calcium sulfate. The total phenol content of berries in the vines treated with the second level of potassium sulfate and calcium sulfate without mineral oil (V_0_K_1_C_1_) showed an increase of up to 42% compared with the control vines and up to 49% rise compared with the vines treated with the second level of mineral oil alone (Fig. 2).

**Fig. 3:**
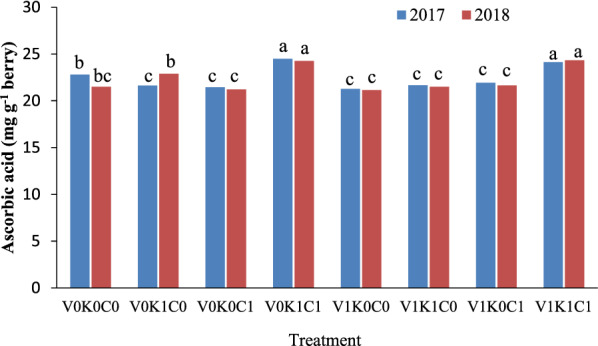
The combination effect of mineral oil, potassium sulfate, and calcium sulfate on berry ascorbic acid content of Sultana grapevine. The means with common letters in each column according to Duncan's test are not significantly different (*P* < 0.05). V_0_ = 0.00% mineral oil, V_1_ = 1.00% mineral oil; K_0_ = 0.00% K_2_SO_4_, K_1_ = 3.00% K_2_SO_4_; C_0_ = 0.00% CaSO_4_, C_1_ = 2.00% CaSO_4_

The ascorbic acid content of berries in the vines treated with the second level of potassium sulfate and calcium sulfate without mineral oil (V_0_K_1_C_1_) was higher than other treatments, however, there was no statistically significant difference with the vines treated with the combination of the second level of all three materials (V_1_K_1_C_1_). Other treatments did not show significant differences with others in terms of ascorbic acid content (Fig. 3).

### Raisins yield

**Fig. 4:**
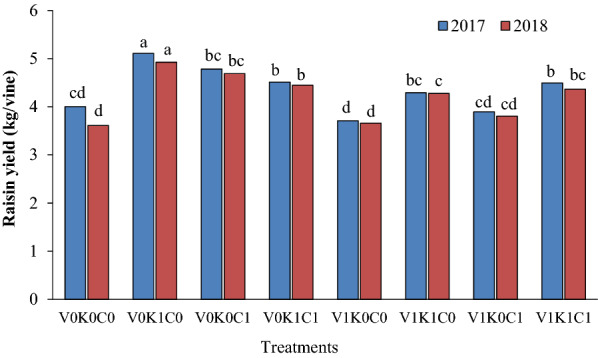
The combination effect of mineral oil, potassium sulfate, and calcium sulfate on produced raisin of Sultana grapevine. The means with common letters in each column according to Duncan's test are not significantly different (*P* < 0.05). V_0_ = 0.00% mineral oil, V_1_ = 1.00% mineral oil; K_0_ = 0.00% K_2_SO_4_, K_1_ = 3.00% K_2_SO_4_; C_0_ = 0.00% CaSO_4_, C_1_ = 2.00% CaSO_4_

The yield of raisins obtained from the vines treated with the second level of potassium sulfate alone (V_0_K_1_C_0_) was higher than other treatments, while the vines treated with the second level of mineral oil alone (V_1_K_0_C_0_) showed less performance in terms of raisin production so that they did not show significant differences with the control vines. In fact, the yield of raisins obtained from the vines treated with the second level of potassium sulfate alone (V_0_K_1_C_0_) was 27% higher than the vines treated with the second level of mineral oil alone (V_1_K_0_C_0_) and 22% higher than that of the control vines (Fig. 4).

## Discussion

Adequate nutrient supply is important especially in the early growing season to provide the necessary structural components for bud bloom as well as successful fertilization of flowers. At the beginning of the season, due to the increase in growth in buds and the predominance of vegetative growth, the concentration of nutrients in plant tissues decreases [30]. In these conditions, elements such as potassium due to high need in the vine or elements such as calcium are not provided enough for the vine due to the slow movement from the soil to the roots and transport in the vessels. Therefore, these conditions are considered as an important factor limiting flowering, fruit set, and final fruit quality in the vineyards [10]. In the present study, the early-season application of potassium sulfate and calcium sulfate in combination with mineral oil affected all the measured indices such as leaf elements concentration, phenological indices, yield, and fruit quality of Sultana grapevine.

The vines treated with potassium sulfate alone or in combination with calcium sulfate showed higher concentrations of nutrients such as leaf potassium, calcium, magnesium, and nitrogen. In grapes, foliar application of potassium sulfate [24, 48], potassium sulfate and urea [27], and calcium sulfate and zinc sulfate [25] increased the concentration of some nutrients in leaves and fruits. Calcium foliar application during 2 to 3 weeks after the full bloom stage effectively increased the calcium content in the fruit tissue of Golden Delicious apple cultivar [31]. In an experiment on pistachio trees, application of mineral oil did not increase phosphorus and potassium content in the leaves, but increased iron, zinc, and manganese content compared with control plants [1]. The combined application of mineral oil and potassium nitrate in pistachios at the silver tip stage (February) increased the nitrogen and potassium content in flower buds compared with the control treatment [23]. In pistachio, leaf iron and zinc content of the trees sprayed with mineral oil was lower than untreated plants [1]. It is possible that mineral oil spray increases the absorption efficiency of some nutrients such as phosphorus, iron, and zinc [23]. Higher levels of elements such as phosphorus in the leaves of the trees sprayed with mineral oil alone or mixed with dietary supplements can be due to the stabilization of nutrients by mineral oil in the leaves [1]. It seems that the combined use of nutrients with mineral oil has a better effect on improving the absorption of elements compared with the use of mineral oil alone. In other words, the combination of these nutrients with mineral oil can increase essential nutrients, which is suitable for the reproductive growth of the plant in the growing season. Genetic and viticultural factors such as fertilization and ecological factors (such as temperature, light, humidity, and soil) have a great impact on the leaf and fruit elements [30]. Potassium and calcium increase photosynthesis and sugar production to enter the target organs [24, 36]. Increased yield, as well as carbohydrate and nitrogen reserves in the plants treated with these nutrients, may increase nutrient uptake by increasing root growth and developing root contact with soil [42]. Witnesses have grown especially in critical stages. On the other hand, adequate potassium supply in the vine, while osmotic regulation in the tissues increases the mobility of the elements, which confirms the findings of the present study.

In the treatments containing mineral oil, especially in combination with the second level of calcium and potassium (V_1_K_1_C_1_), bloom time, berries pea-sized time, and harvest time were delayed by 3, 3, and 6 days compared with control vines, while in the vines treated with a combination of the second level of potassium and calcium (V_0_K_1_C_1_), bloom time, berries pea-sized time, and harvest time were advanced by 5, 4, and 1.50 days, respectively. Foliar application of soybean oil delayed the onset of flowering time and full flowering time in apricots [14]. Also, calcium chloride and calcium nitrate spray in combination have led to a significant increase in the quality and concentration of nutrients in apple fruit [47]. Increasing the concentration of nutrients, especially calcium and potassium in the plants treated with potassium sulfate and calcium sulfate, by increasing the necessary infrastructure for the production of sugars and proteins, paves the way for regulating the flowering time [25]. It seems that an adequate supply of elements such as calcium and potassium, especially in the stage of rapid fruit growth (berries pea-sized) while increasing photosynthesis causes the material to reach the berries and thus increases berry size and cluster weight. On the other hand, mineral oil reduces leaf photosynthesis at the beginning of the season [23], which can affect the completion of phenological and fruit development stages [1].

The highest percentage of fruit set was obtained in the plants treated with 3.00% potassium sulfate, which showed a significant difference with other treatments, and the lowest fruit formation was obtained in the control vines (Table 3), indicating the role of calcium [26] and potassium [20, 24] in fruit set. Potassium can improve the effect of transferring other elements and cause better loading of photosynthetic products such as glucose and sucrose into the phloem vessels during flowering, fertilization, and fruit set [30, 34]. Calcium contributes to auxin activity and is involved in cell division and cell elongation, germination, and pollen tube growth [21]. Calcium is effective in improving flowering, maturation, and transfer of carbohydrates from leaves to fruits [32]. It seems that an adequate supply of nutrients during the plant growth period while increasing the effective pollination period has increased the percentage of fruit set. Calcium and potassium play an important role in many physiological and biochemical processes required for growth and development, including cell division and elongation, flowering and fruit set, which can directly affect the percentage of fruit set [10]. Application of soybean oil in Fakhri cultivar reduced fruit set compared with the control vines [12], which is consistent with the results of the present study. High concentrations of oils, due to the opacity of the buds and the excessive accumulation of respiratory gases within them, cause the death of the buds and subsequently cause thinning and falling of flowers, compared with lower concentrations [11, 17]. On the other hand, the relatively long delay in the bloom date in the vines treated with mineral oil caused the pollination stage and fertilization of flowers at a temperature above the optimal level, and this somewhat reduces the success in practice [12, 13]. The effect of a high concentration of soybean oil on reducing the percentage of fruit set and subsequent yield reduction in different grape cultivars [12, 13], peach [17], and pistachio [1] have been reported, which confirmed the negative effect of horticultural oils on flowering and fruit set.

Regarding the biophysical indices of the cluster, it was found that the vines treated with V_1_K_1_C_1_ had more cluster weight, berry weight, fruit, and raisins yield than other treatments. In the Thomson Seedless cultivar, the application of calcium chloride in leaves and soil has led to an increase in berry size and cluster weight compared with control vines [10]. Also, foliar spraying of Washington Novell orange trees with 1.00% calcium chloride increased fruit weight, number, and total yield [4]. Also, foliar application of calcium, calcium, chelate, and boron in Valencia orange cultivar significantly increased fruit weight, number of fruits per tree, and final yield compared with the control [8], which confirmed the findings of the present study. Berry weight is one of the important quantitative indicators in fresh grapes that play an important role in its quality and marketability. In the previous study, foliar application of potassium sulfate increased the weight of berries and finally the weight of grape clusters [24]. Potassium increases osmotic pressure and water uptake, carbohydrate metabolism, and assimilate transport in the phloem, which in the absence of potassium, disrupts the transport of substances and their accumulation in the leaves, leading to lack of allocation to fruits and weight loss [30]. Application of a combination of calcium sulfate and zinc sulfate [26], potassium sulfate [24] in Bidaneh Sefid grapes increased yield and yield components such as berry weight, number of berries, and volume of berries. Foliar application of potassium sulfate in grapes increased berry weight, panicle weight, berry length, fruit sugar, and fruit acidity [48]. Although potassium does not form functional molecules or plant structures, it is involved in numerous biochemical and physiological processes critical to plant growth, yield, and fruit quality [32].

In line with the present results, the use of other horticultural oils such as soybean oil in grapes [12, 16] and peaches [17] has reduced yield compared with control plants, which confirms the results of the present study. Also Dami and Beam [13] during the study of the effect of soybean oil on the delay budburst in grapevine, reported that with increasing the oil concentration, the weight of the cluster decreases compared with the control, but due to the thinning properties of soybean oil, the weight of single berries will be increased.

The amount of berries quality such as TSS, pH, total phenol, and ascorbic acid in the vines treated with the second level of potassium sulfate and calcium sulfate without mineral oil (V_0_K_1_C_1_) showed an increase compared with other treatments. The use of nutrients such as potassium [24, 35] in grapes and calcium in kiwifruit [22] has led to an increase in TSS. In orange trees, foliar application of chelate, calcium, and chelate significantly increased the TSS and decreased the TA of the fruit compared with the controls [4, 8], which confirms the findings of the present study. The results of foliar spraying of potassium sulfate in grapes of Crimson Seedless cultivar have shown that foliar application effectively reduces acid content [20]. In Valencia orange cultivar, foliar application of calcium significantly increased the total soluble solids and decreased the titratable acidity of the fruit compared with the control treatment [4, 8], which confirms the findings of the present study. Based on the result of the present study, the lowest pH and TSS of fruit were in the control vines and the vines treated with the second level of mineral oil alone. In the study of Chayani et al. [12], the lowest berry pH and phenol content and the highest acid content were obtained with 10.00% soybean oil treatments, and its combination with naphthalene acetic acid and the lowest acid content and the highest pH were observed in the control and single foliar application of naphthalene acetic acid. Dormant oils has an effect on fruit quality, chemical depending on the climatic conditions of the region, time of application and plant species [13].

It has been reported that the use of soybean oil with a concentration of 10.00% and naphthalene acetic acid with concentrations of 1000, 500, and 2000 mg/l on Edelweiss grapes did not change the chemical properties of the fruit compared with the control and only reduced the berry juice pH [41]. In his study, fruit quality indices were greatly affected by foliar application, especially with 10.00% soybean oil. Due to the relatively large effect of this compound on the delay in bud opening date and the fact that the fruit of each treatment was harvested on the same date, the change in fruit quality is probably due to receiving a different heat unit and affected by the climate of the region and the type of cultivar and therefore, the role of oils on berry quality such as phenol content is not specific [12]. The phenol content in the fruit is more affected by the amount of maturity and environmental conditions of the plant. Foliar application of potassium [35] has increased total phenolics in the fruit, which is consistent with the results of the present study. Potassium stimulates photosynthetic activity and increases the transport of sugars to the fruit, which indirectly accumulates sugars in the biology of phenolic compounds during ripening and is closely related to the presence of carbohydrates in the berries [15]. The increase in total phenol content observed in this experiment under the influence of calcium and potassium treatments indicates the key role of these ions indirectly in the biology of phenolic compounds.

The highest concentrations of ascorbic acid were related to plants treated with the second level of potassium sulfate and calcium sulfate without mineral oil (V_0_K_1_C_1_). In a study on grapes, the highest amount of ascorbic acid (3.69 mg/100 g) was found in the vines treated with 1.50% potassium sulfate alone [35]. Also, foliar application of potassium sulfate in Rasheh grape cultivar increased the ascorbic acid of seeds [48], which is consistent with the results of the present study. Ascorbate can enter the tubers by oxidation of glucose in the tubers or by transfer from the leaves by the phloem vessels, and sometimes they may even be produced inside the phloem vessels [30].

The effect of foliar application of potassium sulfate on Rasheh [48] and Bidaneh Sefid [35] grapevine cultivars increased soluble solids and dry matter content. The ability of potassium to accumulate more dry matter is one of the reasons for increasing the yield of raisins and increasing the conversion ratio of grapes to raisins in the vines treated with V_0_K_1_C_1_.

## Conclusion

Bloom time, berries pea-sized time, and harvest time were advanced by 5, 4, and 1.50 days, respectively in the vines treated with a combination of the second level of potassium and calcium (V_0_K_1_C_1_) compared with control vines. Moreover, the vines treated with V_1_K_1_C_1_ had higher cluster weight, berry weight, fruit yield, and raisins yield than other treatments. Also, the highest berry quality, including total soluble solids, titratable acidity, and total phenol content were obtained in the vines treated with V_0_K_1_C_1_. Therefore, the combination of nutrients with mineral oil can alleviate the adverse effect of mineral oil solely on berry quality and phenological indices in vineyards.

## Data Availability

When the Editors want, we submit data.
